# Effects of Brucine on the OPG/RANKL/RANK Signaling Pathway in MDA-MB-231 and MC3T3-E1 Cell Coculture System

**DOI:** 10.1155/2017/1693643

**Published:** 2017-09-26

**Authors:** Ruixian Wu, Qian Li, Xiaohua Pei, Kefei Hu

**Affiliations:** ^1^Third Clinical Medical College, Beijing University of Chinese Medicine, Beijing 100029, China; ^2^The Third Affiliated Hospital, Beijing University of Chinese Medicine, Beijing 100029, China; ^3^Beijing Yuan Lai Health Technology Development Co. Ltd., Beijing 100035, China

## Abstract

The present study examined the effects of brucine on the OPG/RANKL/RANK signaling pathway for exploring the mechanism of brucine suppression of bone metastasis in breast cancer. MDA-MB-231 breast cancer cells and mouse osteoblast MC3T3-E1 cells were cocultured to mimic the breast cancer bone metastasis microenvironment* in vitro*. qRT-PCR and Western blotting were used to detect the expressions of OPG and RANKL at the mRNA and protein levels, respectively, in brucine-treated cultures and they were compared to those in untreated cultures. We aimed to understand the effect of brucine on the entire OPG/RANKL/RANK signaling pathway after analyzing these effects. Results showed that brucine treatment significantly increased both the OPG mRNA/RANKL mRNA expression ratio and the OPG protein/RANKL protein ratio in cocultures compared to those in untreated cocultures (*P* < 0.01). Brucine, therefore, plays a regulatory role in the OPG/RANKL/RANK signaling pathway, suggesting that it can indirectly control osteoclasts by regulating the expression and secretion of OPG and RANKL in osteoblast cells, thereby inhibiting the differentiation and bone resorption function of osteoclasts.

## 1. Introduction

Breast cancer is the leading cause of cancer-related mortality in women, with half million deaths annually worldwide [1]. More than 90% of these deaths are caused by metastasis [2], as approximately 65–75% of the patients with metastatic breast cancer develop bone metastases [3, 4]. Breast cancer bone metastasis is closely associated with osteoclastogenesis and osteolytic bone metastasis [5]; however, the precise molecular mechanisms are not fully understood.

Semen Strychni was first documented in the Compendium of Materia Medica and was believed to be capable of removing tissue masses owing to its ability to promote blood circulation and granulation and remove necrotic tissue. For these reasons, it has been used in cancer treatment for a long time. Brucine is the major active ingredient in Semen Strychni. Extensive research has been conducted in the past few years to investigate the antineoplastic effect of brucine [6, 7]. Several researchers have shown that Semen Strychni has a therapeutic effect in hepatoma cell lines (e.g., SMMC-7221, HepG2, and H22), breast cancer cell lines (e.g., MDA-MB-231 and MCF-7), and hematological tumor cell lines (e.g., K562 and U266), among others. Some researchers have also suggested that brucine might inhibit the growth of bone metastases in breast cancer of nude mice and alleviate bone destruction [8, 9]. Therefore, the effect of brucine on bone metastases in breast cancer has attracted attention.

The observation that the OPG/RANKL/RANK system plays a vital role in osteoclastogenesis has been a significant breakthrough in the field of bone physiology [10–12]. The OPG/RANKL/RANK system plays a critical role in maintaining bone balance, which determines whether osteolytic metastasis would be initiated. In this study, we examined the key factors in this system, namely, OPG and RANKL, in an* in vitro* coculture model using the MDA-MB-231 breast cancer and the mouse osteoblast MC3T3-E1 cell lines. Specifically, we examined the interaction between the breast cancer cells and osteoblasts in a microenvironment that mimicked bone metastases in breast cancer and assessed the effect of brucine, using alterations in the mRNA and protein levels of OPG and RANKL as readouts.

## 2. Materials and Methods

### 2.1. Cells

The human breast cancer cell line MDA-MB-231 and mouse osteoblast cell line MC3T3-E1 (Shanghai Cell Bank of the Chinese Academy of Sciences, Shanghai, China) were used.

### 2.2. Reagents

Fetal bovine serum (HyClone, Logan, Utah, USA), trypsin (Gibco, Grand Island, NY, USA), *α*-modified Eagle's medium (MEM) (Life Technologies Corporation, NY, USA, lot number 1406320), 2x Taq PCR Master Mix (dye included), Taq polymerase, SYBR Green PCR Master Mix (Invitrogen Corporation, Carlsbad, CA, USA), DEPC (diethyl pyrocarbonate), DEPC-treated water (DNase/RNase-free double distilled H_2_O) (Amresco, Solon, OH, USA), anti-rabbit IgG HRP-linked (7074S, Cell Signaling Technology, Danvers, MA, USA), rabbit anti-goat IgG-HRP (sc-2768, Santa Cruz, USA), and Western blot marker (color predyed) (Thermo Fisher Scientific) were used.

### 2.3. Drugs

Brucine (molecular formula: C_23_H_26_N_2_O_4_) was purchased from the National Institute for Food and Drug Control (lot number 110706-201306; purity: 91.7%). Zoledronic acid injection was manufactured by Novartis Pharma Schweiz AG (catalog number S0051; specification: 100 mL; 5 mg).

### 2.4. Instruments

Eco Real-Time PCR system (Illumina, San Diego, CA, USA), high-speed centrifuge, pipettes (range: 2.5 *μ*L to 1000 *μ*L) (Eppendorf, Hamburg, Germany), VE-180 vertical electrophoresis unit, VE-186 Trans-Blot transfer unit (Tanon Science and Technology, Shanghai, China), vortex shaker (model: HYQ-3110; Crystal Technology & Industries, Dallas, Texas, USA), microplate reader (model: ELX800; BioTek Laboratories, Vermont, USA), and ultrasonic cleaner (model: SB-5200DTDN; Scientz, Ningbo, China) were used.

### 2.5. Experimental Groups

For the model group, the human breast cancer cell line MDA-MB-231 and the mouse osteoblast MC3T3-E1 cell line were cocultured in the presence of an induction medium (*α*-MEM) containing *β*-glycerophosphate (10 mmol/L) and L-ascorbic acid (50 *μ*g/mL). The control group was the same as the model group minus the MDA-MB-231 breast cancer cells. The brucine group with 0.02 mmol/L is a model group treated with 0.02 mmol/L brucine. The brucine group with 0.04 mmol/L is a model group treated with 0.04 mmol/L brucine. The brucine group with 0.08 mmol/L is a model group plus 0.08 mmol/L brucine. The positive control drug group is a model group plus 10 *μ*mol/L zoledronic acid.

### 2.6. Coculture of MDA-MB-231 and MC3T3-E1 Cells

The MC3T3-E1 mouse osteoblast cell line was cultured in *α*-MEM as a single cell suspension. The cell concentration was adjusted to 1 × 10^6^/mL and the suspension was inoculated into a T-75 culture flask and cultured until the MC3T3-E1 cells reached approximately 80%–90% confluence. The medium was then replaced with *α*-MEM induction medium containing *β*-glycerophosphate (10 mmol/L) and L-ascorbic acid (50 *μ*g/mL). Six days after induction, the breast cancer cell MDA-MB-231, which had been grown as a single cell suspension and adjusted to a cell concentration of 5.6 × 10^4^/mL, was added directly to the culture flask. The cells were then cocultured for seven days with different experimental media as described above. The media were replaced after every two days.

### 2.7. Quantitative Real-Time Polymerase Chain Reaction (qRT-PCR)

Total RNA was extracted using the TRIzol kit (Life Technologies, NY, USA) according to the manufacturer's instructions. A small amount of RNA was removed to determine its optical density (OD) value and the remainder was stored at −80°C.

The 20 *μ*L PCR reaction system included 1 *μ*L primer, 11 *μ*L nuclease-free ultrapure water, 4 *μ*L 5x reaction buffer, 1 *μ*L RiboLock™ RNA enzyme inhibitor (20 U/*μ*L), 2 *μ*L 10 mM dNTP mix, and 1 *μ*L RevertAid™ M-MuLV reverse transcriptase (200 U/*μ*L) (Thermo Fisher Scientific, Waltham, MA, USA).

Fluorescent quantitative PCR was conducted as follows: in a PCR tube, the cDNA template (1 *μ*L), upstream primer (1 *μ*L, 10 *μ*M), downstream primer (1 *μ*L, 10 *μ*M), SYBR green (10 *μ*L), and double-distilled H_2_O (7 *μ*L) were mixed together to obtain a total reaction volume of 20 *μ*L. PCR was performed using the following cycle: 50°C for 2 min, 95°C initial denaturation for 10 min, 95°C denaturation for 15 s, and 60°C annealing for 30 s, for a total of 40 cycles. The primer sequences used are shown in Table 1.

### 2.8. Western Blotting

Drugs were added to cocultures of MDA-MB-231 and MC3T3-E1 cells for 24 h as described above. Total protein was extracted from the cells using radioimmunoprecipitation assay (RIPA) lysis buffer (Solarbio, Beijing, China). Equal amounts of protein extracts were then separated using 10% sodium dodecyl phosphate-polyacrylamide gel electrophoresis (SDS-PAGE) (Gibco, Grand Island, USA) and transferred onto a polyvinylidene fluoride (PVDF) membrane (0.45 *μ*m) (Millipore, Bedford, MA, USA). The membranes were blocked with 5% w/v nonfat dry milk (Gibco, Grand Island, USA) dissolved in Tris-buffered saline (Amresco, Solon, OH, USA) plus Tween-20 (TBS-T) (Gibco, Grand Island, USA) at 25°C for 1 h, followed by overnight incubation with primary antibodies at 4°C. The primary antibodies for immunoblotting were anti-OPG antibody (P-17) (sc-21038, Santa Cruz, CA, USA), anti-RANKL antibody (ab124797, Abcam, Cambridge, UK), and anti-*β*-actin antibody (N-21) (sc-130657, Santa Cruz, USA). After washing with TBS-T (CoWin Biotech, Beijing, China), the membranes were incubated with HRP-labeled secondary antibodies (Cell Signaling Technologies, Danvers, MA, USA) for 1 h at 25°C. The membranes were analyzed using a protein visualizer ECL (Tanon Science and Technology, Shanghai, China).

### 2.9. Statistical Analysis

All experiments were repeated at least thrice. SPSS 20.0 data analysis software was used for statistical analysis and processing. Kruskal-Wallis *H*-test was employed for the nonparametric test of two or more independent samples. All data are expressed as the mean ± standard deviation. *P* values less than 0.05 (*P* < 0.05) were considered statistically significant.

## 3. Results

### 3.1. Effect of Brucine on OPG and RANKL mRNA Levels

**Figure 1:**
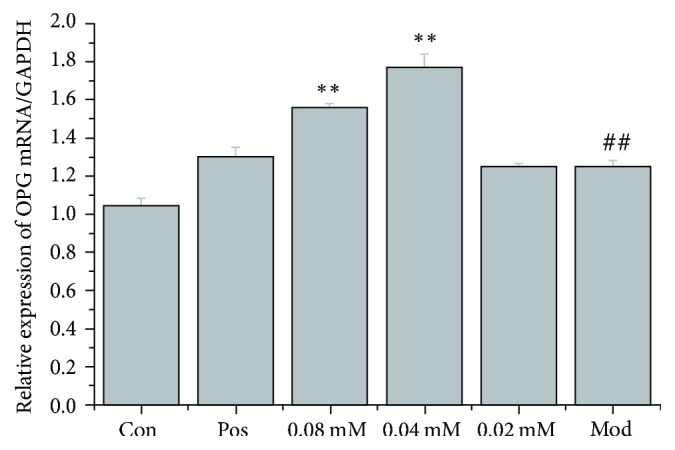
Comparison of OPG mRNA levels in different experimental groups.

**Figure 2:**
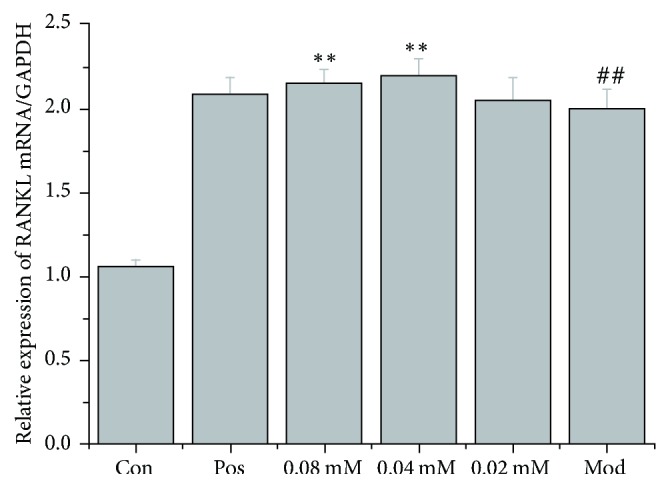
Comparison of RANKL mRNA levels in different experimental groups.

qRT-PCR was used to measure OPG and RANKL mRNA levels in cocultures of the human breast cancer cell line MDA-MB-231 and the mouse osteoblast MC3T3-E1 cell line with or without brucine. The model group's OPG and RANKL mRNA levels were significantly higher than those of the control group (*P* < 0.01). Brucine (0.04 and 0.08 mmol/L) increased OPG and RANKL mRNA levels significantly compared to the model group (*P* < 0.01 or *P* < 0.05), as shown in Figures 1 and 2.

Figures 1 and 2 show comparison of OPG and RANKL mRNA levels in different experimental groups. qRT-PCR was used to measure the OPG and RANKL mRNA levels. MDA-MB-231 and MC3T3-E1 cells were cocultured for 7 days; different doses of brucine (0.02, 0.04, and 0.08 mmol/L) and 10 *μ*mol/L zoledronic acid were given (Figure 1, ^##^*P* < 0.01, compared to the control group; ^*∗∗*^*P* < 0.01, compared to the model group; Figure 2,  ^##^*P* < 0.01, compared to the control group; ^*∗∗*^*P* < 0.05, compared to the model group).

### 3.2. Effect of Brucine on OPG mRNA/RANKL mRNA Ratio

**Figure 3:**
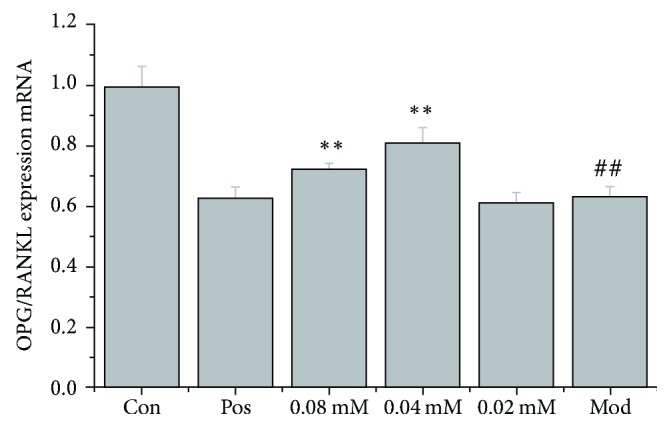
Comparison of the OPG mRNA/RANKL mRNA ratio in different experimental groups.* Notes*. ^##^*P* < 0.01, compared to the control group; ^*∗∗*^*P* < 0.01, compared to the model group.

Since osteoblasts secrete both OPG and RANKL, which are required for bone protection and bone destruction, respectively, the ratio of OPG/RANKL more accurately reflects the balance between bone absorption and bone reconstruction. Therefore, we analyzed and compared the ratio of OPG mRNA/RANKL mRNA in the different experimental groups. Results showed that the OPG mRNA/RANKL mRNA ratio of the model group was significantly lower compared to that of the control group (*P* < 0.01). Brucine (0.04 and 0.08 mmol/L) increased the OPG mRNA/RANKL mRNA ratio significantly compared to that in the model group (*P* < 0.01) (Figure 3).

### 3.3. Effects of Brucine on OPG and RANKL Protein Levels

**Figure 4:**
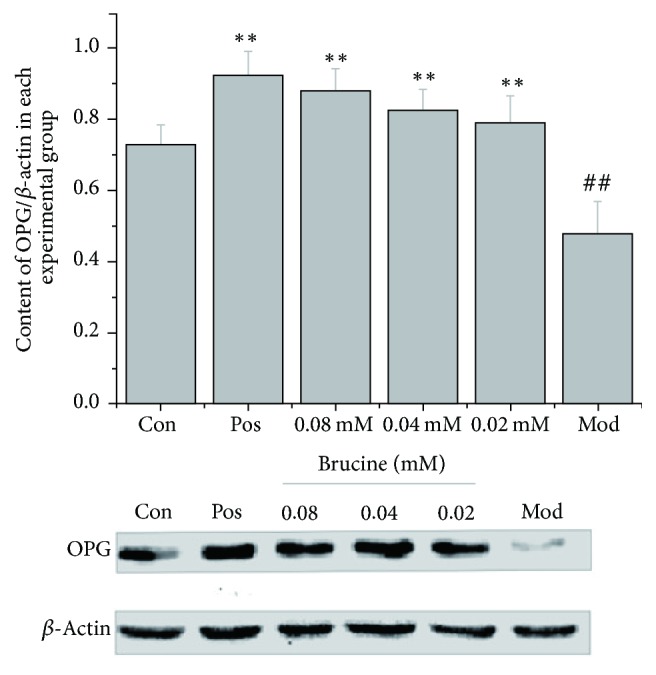
Comparison of OPG protein levels in different experimental groups.

**Figure 5:**
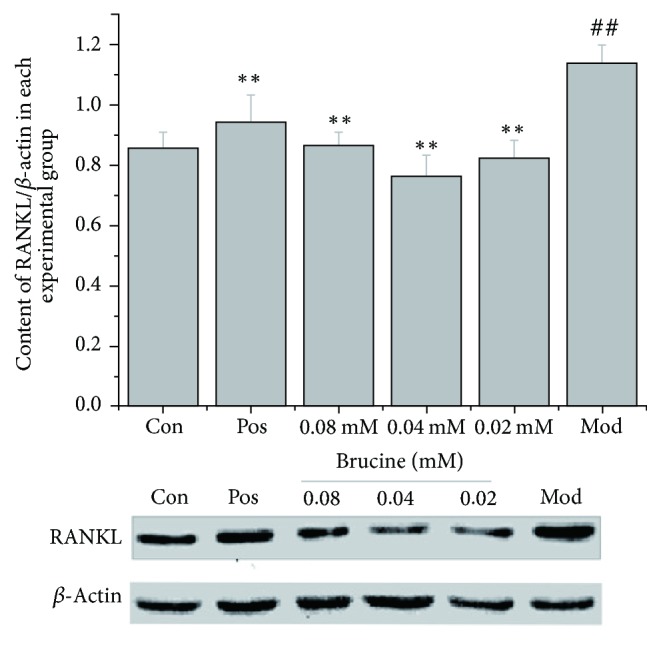
Histogram showing RANKL protein level in different experimental groups.

Western blot was used to measure the OPG and RANKL protein levels in different experimental groups. Results showed that OPG level was significantly lower in the model group compared to that in the control group (*P* < 0.01). In contrast, the model group's RANKL level was significantly higher than that of the control group (*P* < 0.01). Brucine (0.02, 0.04, and 0.08 mmol/L) and the positive control drug zoledronic acid significantly increased OPG levels and decreased RANKL levels, compared to those in the model group (*P* < 0.01) (Figures 4 and 5).

Figures 4 and 5 show comparison of OPG and RANKL protein levels in different experimental groups. The Western blot method was used to measure the OPG and RANKL protein expression levels. MDA-MB-231 and MC3T3-E1 cells were cocultured for 7 days; different doses of brucine (0.02, 0.04, and 0.08 mmol/L) and 10 *μ*mol/L zoledronic acid were given (Figure 4, ^##^*P* < 0.01, compared to the control group; ^*∗∗*^*P* < 0.01, compared to the model group; Figure 5,  ^##^*P* < 0.01, compared to the control group; ^*∗∗*^*P* < 0.01, compared to the model group).

### 3.4. Effects on the OPG/RANKL Protein Ratio

**Figure 6:**
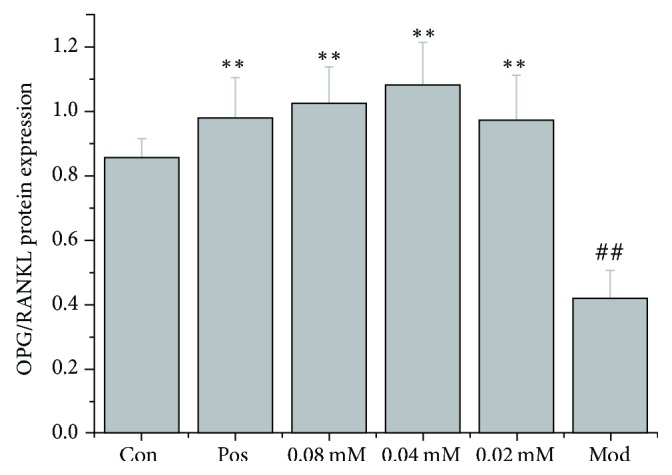
Comparison of the OPG protein /RANKL protein ratio in different experimental groups.* Notes*. ^##^*P* < 0.01, compared to the control group; ^*∗∗*^*P* < 0.01, compared to the model group.

Similar to the analysis conducted for mRNA levels, we analyzed and compared the OPG protein/RANKL protein ratio in different experimental groups. Results showed that the OPG protein/RANKL protein ratio of the model group was significantly lower compared to that of the control group (*P* < 0.01). Brucine (0.02, 0.04, and 0.08 mmol/L) and the positive control drug zoledronic acid significantly increased the OPG/RANKL protein ratio compared to that in the model group (*P* < 0.01) (Figure 6).

## 4. Discussion

Breast cancer, along with lung and prostate cancers, is likely to metastasize to the bone [3]. Bone metastases worsen the prognosis, as chances of survival decrease and the quality of life of the patient dramatically deteriorates, with a clinical outcome characterized by intractable pain, nerve compression syndromes, increased risk of fractures, and hypercalcemia [13]. Certain drugs, including estrogen, bisphosphonates, teriparatide, denosumab, and synthetic calcitonin, have been used for the treatment of osteolysis. Most of these drugs have serious limitations or side effects, such as osteonecrosis, osteosarcoma, thromboembolism, and esophageal irritation [14–16]. Brucine is a bitter alkaloid extracted from the* Strychnos nux-vomica* tree, found in Southeast Asia. Several studies have shown that brucine is an effective agent for the treatment of breast cancer [17]. Intriguingly, brucine has been found to inhibit bone metastasis in breast cancer [18], vascular endothelial growth factor (VEGF) expression, and angiogenesis [8, 19]; however, its precise mechanism of action remains unknown.

The process of bone metastasis in breast cancer is complex and arises due to the interaction between multiple cells in the bone microenvironment which communicate through the release of numerous cytokines. In this process, the OPG/RANKL/RANK signaling pathway is considered to be critical for adjusting bone metabolic balance. As a member of the tumor necrosis factor (TNF) superfamily of ligands, RANKL, which is expressed primarily by osteoblasts and bone marrow stromal cells and occurs membrane-bound on the surface of these cells, plays an important role in the activation of lymphocytes, dendritic cells, and osteoclasts [20, 21]. RANK, the receptor for RANKL, is found on the cell membrane of lymphocytes, activated T cells, B cells, osteoclasts, and other cell types and is important for osteolysis and growth of the lymph node [22, 23]. OPG is a member of the tumor necrosis factor receptor superfamily, which inhibits the differentiation of osteoclasts, and a reduction in its expression has been considered to be a key factor in the occurrence and development of bone metastases in breast cancer. Although OPG is widely expressed in a variety of tissues, it is functional only in the bone. Studies showed that osteoblasts regulate the differentiation of osteoclasts via the secretion and expression of OPG and RANKL [24]. Thus, the levels of OPG, RANKL, and RANK in the local microenvironment of bone tissues are considered as defining biological factors, which mediate a variety of molecules to induce the production and function of osteoclasts, and are also key determinants of the balance between bone destruction and formation [25–27].

Our previous study demonstrated that brucine inhibited osteoclastogenesis by suppressing the Jagged1/Notch1 signaling pathways [28]. In the current study, we demonstrate that brucine (0.04 and 0.08 mmol/L) significantly increases both OPG and RANKL mRNA levels compared to those in the model group. Brucine also increases the OPG mRNA/RANKL mRNA ratio in cocultures of the human breast cancer cell line MDA-MB-231 and the mouse osteoblast MC3T3-E1 cell line. Different doses of brucine and the positive control drug zoledronic acid significantly increased OPG protein levels and decreased RANKL protein levels. However, brucine and zoledronic acid also significantly increased the OPG protein/RANKL protein ratio. These results suggest that brucine may regulate the bone metabolic balance by regulating OPG/RANKL/RANK signaling pathways.

Osteoclasts are giant multinuclear cells derived from the monocyte-macrophage lineage. Osteoclast differentiation from precursor cells can be induced by RANKL, which also controls the survival and function of mature osteoclasts. Therefore, RANKL has been used in studies on differentiation and function of osteoclasts. Comparison of the experimental data from the model and control groups showed that the introduction of the breast cancer cells decreased the OPG/RANKL ratio, which might have stimulated osteoclast activation and differentiation via increase in RANK-RANKL numbers and intracellular signal transduction, thereby disrupting bone metabolic balance. Comparison of the brucine data from the model groups showed that the introduction of the brucine increased the OPG/RANKL ratio. We conclude that brucine inhibited the OPG/RANKL/RANK signaling pathway by regulating both mRNA and protein levels. On one hand, brucine directly affected osteoclast differentiation, while, on the other hand, it inhibited osteoclast differentiation and maturation via indirect regulation of osteoclast function by osteoblasts.

## 5. Conclusions

In conclusion, brucine appears to play a regulatory role in the OPG/RANKL/RANK signaling pathway, suggesting that it can indirectly control osteoclasts possibly by regulating the OPG and RANKL levels secreted by osteoblast cells, thereby inhibiting the differentiation and bone resorption functions of osteoclasts. Further studies are required for verifying whether brucine can inhibit bone metastases of breast cancer* in vivo* and whether OPG and RANKL levels are related to bone metastases in brucine-treated breast cancer patients.

## Figures and Tables

**Table 1:** Primer sequences.

Name	Primer sequence
OPG upstream primer	5′-ATGGACAACCCAGGAAACCC-3′
OPG downstream primer	5′-GTAGGTGCCAGGAGCACATT-3′
RANKL upstream primer	5′-ATGATGGAAGGCTCATGGTTGG-3′
RANKL downstream primer	5′-CAGCATTGATGGTGAGGTGTG-3′
GAPDH upstream primer	5′-AGCCTTCCTTCTTGGGTATG-3′
GAPDH downstream primer	5′-GGTCTTTACGGATGTCAACG-3′
